# Enteric Methane Emissions from Dairy–Beef Steers Supplemented with the Essential Oil Blend Agolin Ruminant

**DOI:** 10.3390/ani13111826

**Published:** 2023-05-31

**Authors:** Gemma A. Miller, Jenna M. Bowen, Richard J. Dewhurst, Beatrice Zweifel, Katrin Spengler, Carol-Anne Duthie

**Affiliations:** 1 Beef and Sheep Research Centre, Scotland’s Rural College, West Mains Road, Edinburgh EH9 3JG, UK; 2 Dairy Research and Innovation Centre, Scotland’s Rural College, West Mains Road, Edinburgh EH9 3JG, UK; 3 Agolin, SA, Route de la Picarde 20, 1145 Bière, Switzerland

**Keywords:** Agolin Ruminant, enteric methane, essential oils, greenhouse gas mitigation, methane inhibitor, dairy–beef, cattle, ruminant

## Abstract

**Simple Summary:**

Methane is a greenhouse gas that substantially contributes to climate change. Agriculture is the largest source of methane globally, and more specifically, methane produced by ruminants during feed digestion (32% of global methane emissions). Essential oils have properties that may reduce the amount of methane produced by ruminants. This study tested the effectiveness of an essential oil product in reducing methane emissions from dairy–beef cattle. Methane emissions were measured from individual animals by confinement in respiration chambers. Results showed that although there was no difference in the mass of methane produced, the yield (grams of methane per kilogram of feed dry matter consumed) was lower in the animals receiving the essential oils. This reduced methane yield was caused by control animals consuming less feed during methane measurement periods. The same reduction in feed intake was not observed in the treatment animals. This suggests that animals supplemented with essential oils were less affected by confinement in respiration chambers than control animals.

**Abstract:**

Agriculture is the largest source of methane globally, and enteric methane accounts for 32% of methane emissions globally. Dairy–beef is an increasingly important contributor to the beef industry. The objective of this study was to investigate if supplementation with a blend of essential oils (Agolin Ruminant) reduced enteric methane emissions from dairy-bred steers. Methane was measured from thirty-six Holstein Friesian steers (18 control and 18 treatment) in open-circuit respiration chambers, at three time-points relative to the introduction of Agolin Ruminant: (i) −3 (pre-additive introduction co-variate), (ii) 46 days after introduction, and (iii) 116 days after introduction. A significantly lower methane yield was observed in treated animals compared to control animals at both 46 days (*p* < 0.05) and 116 days (*p* < 0.01) after the introduction of Agolin Ruminant, although there was no difference in methane production (g/day). Control animals appeared to be more affected by isolation in respiration chambers than animals receiving Agolin Ruminant, as indicated by a significant reduction in dry matter intake by control animals in respiration chambers.

## 1. Introduction

The Global Methane Pledge was launched at the United Nations Framework Convention on Climate Change’s Conference of the Parties in 2021. The goal of the pledge is to reduce global methane emissions by 30% by 2030 (relative to 2020 emissions levels), and it has been signed by 150 countries. Methane is a powerful greenhouse gas with a global warming potential 27.2 times greater than that of carbon dioxide over a 100-year period [1]. Agriculture is the largest source of methane (CH_4_) emissions globally, accounting for 40% of emissions, with enteric CH_4_ alone accounting for 32% [2]. A variety of dietary manipulations have been proposed to reduce enteric CH_4_ emissions from cattle [3]. One such manipulation is supplementation with essential oils. Essential oils are anti-microbial, antioxidant, and anti-inflammatory in nature. They may disrupt cell membranes, reducing the total microbial population of the rumen, or deactivate microbial enzymes, reducing the activity of the microbial population, thus reducing the production of methane [3]. Agolin Ruminant (AR, Agolin SA, Switzerland) is a commercial feed additive that is a blend of essential oils containing coriander oil, geraniol, and eugenol.

Dairy–beef is an increasingly important contributor to the beef industry [4,5] and has a lower emissions intensity than suckler beef as emissions associated with the cow are allocated to dairy production [6]. The emissions intensity of dairy–beef could be driven even lower through dietary manipulation. Agolin Ruminant has shown the potential to reduce methane emissions from lactating dairy cows while improving milk production when supplemented for periods longer than four weeks [7]. However, the efficacy of nutritional strategies to reduce methane emissions may vary depending on the diet offered, production level, sex, and physiological state of the animal [7,8]. Agolin Ruminant has not yet been tested for its efficacy in reducing enteric methane emissions from finishing dairy-bred steers. Therefore, the research question addressed in this study was: is Agolin Ruminant effective at reducing enteric CH_4_ emissions from dairy-bred steers over short time periods (~7 weeks after introduction) and over a finishing period (~17 weeks after the introduction of AR)? We hypothesized that dairy-bred steers supplemented with AR would produce less enteric methane than control steers at both time points.

## 2. Materials and Methods

### 2.1. Experimental Design

This study was conducted at SRUC’s Beef and Sheep Research Centre. The experiment was approved by the Animal Experiment Committee of SRUC and was conducted in accordance with the requirements of the UK Animals (Scientific Procedures) Act 1986.

Thirty-six Holstein Friesian steers (average body weight (BW) 294 ± 6.7 kg, aged 10–12 months) were selected from a wider group of cattle. They were selected to represent the range of BWs and origin farms within the group. Selected cattle were allocated into six blocks balanced for BW and the farm of origin. Animals were paired by BW and farm of origin within the block, and one from each pair was randomly assigned to the treatment group and its pair to the control group. Animals were allocated to one of six respiration chambers so that the allocation was balanced for block and treatment, with one animal from each block entering the chambers each measurement week. Animals were housed in group pens for treatment when not in the respiration chamber facility.

There were three CH_4_ measurement periods: (i) baseline (measured over 72 h prior to the introduction of AR), (ii) 46 days after the introduction of AR, and (iii) 116 days after the introduction of AR. Animals were returned to the same chamber at each period to account for between-chamber effects.

### 2.2. Basal Diet

All animals were offered a 50:50 grass silage:concentrate diet (dry matter basis) at 1.05 timed average daily intake using electronic feeders (HOKO, Insentec, Marknesse, The Netherlands). Daily fresh weight intakes and dry matter intakes (DMI) were recorded for each animal. The ingredient and chemical compositions of the experimental diets are given in Table 1.

### 2.3. Treatment Diet

The active ingredients in AR are coriander seed oil, eugenol, geranyl acetate, and geraniol. The introduction of AR was staggered, with animals in the treatment group beginning to receive AR on the day they exited the respiration chambers after the baseline measurement. The AR was incorporated into the treatment diet premix, prepared in batches of 530 kg as required. The concentrated AR was diluted to 5 L (4888 mL water to 112 mL additive) in a pump action sprayer. The solution was then evenly sprayed onto 50 kg of dark grains in a cement mixer as it turned. This ensured that the solution was absorbed evenly by the grains. The treated grains were then mixed with the remaining dark grains (140 kg), barley (290 kg), and minerals/molasses (40 kg) using a Keenan feeding wagon.

### 2.4. Respiration Chambers

Methane measurements were undertaken in six indirect open-circuit respiration chambers. The concentration of CH_4_ in air samples exhausted from the respiration chambers were measured by infra-red absorption spectroscopy (MGA3000; Analytical Development Company Limited, Amersham, UK). Animals remained in the chambers for 72 h, with the final 48 h of gas concentration measurements used in the data analysis. The method of gas concentration measurement in the respiration chambers is described in detail in [9]. Dry matter intake (kg/day) was recorded in each chamber using electronic feeders (HOKO, Insentec, Marknesse, The Netherlands).

### 2.5. Performance Recording

Between the second and third CH_4_ measurements, there was a 56-day performance recording period. The start of performance recording was staggered, with measurement of animals beginning when they exited the respiration chambers after the 46-day methane measurement; this was to ensure that animals had been receiving AR for the same length of time for each measurement throughout the trial. Individual DMI (kg/day) was recorded for each animal using the same type of HOKO electronic feeders used in the group-housed pens, and BW was measured weekly using a calibrated weigh scale before the fresh feed was offered.

### 2.6. Calculations and Data Analysis

Dosages of AR were calculated based on records of actual masses of feed ingredients weighed into the wagon during total mixed ration formulation each day and corrected for the individual animal’s fresh weight intake on the same day.

Methane emissions measurements at 46 and 116 days after introduction of AR were analyzed by analysis of variance (ANOVA), including the effects of block, chamber, and treatment, and including baseline CH_4_ emission as a covariate. Where appropriate, data were log-transformed prior to analysis to satisfy the assumption of normal distribution (as determined using a Shapiro–Wilk test [10]). The assumption of homogeneity in the data was satisfied as determined using Levene’s test for homogeneity of variance [11]. All data were analyzed in R (R Core Team, 2017) using the lme4 and car packages. 

The feed conversion ratio (FCR) was calculated by dividing average DMI by average daily gain (ADG; the slope of the regression of weekly weights) over the 56-day feed efficiency recording period. One animal was removed from the FCR analysis because lameness during the feed efficiency recording period affected DMI. Feed conversion ratio data were analyzed by ANOVA, including the effects of block, treatment, and BW halfway through the feed efficiency recording period (mid-BW).

## 3. Results

The average estimated dosage of AR consumed across the study period was 1.1 ± 0.1 mL head^-1^ day^-1^.

### 3.1. Methane Emissions

Methane yield in the treatment group did not significantly change between measurement periods but had significantly lower CH_4_ yield than the control group at both 46 (*p* < 0.05) and 116 days (*p* < 0.01) after introduction (Table 2). These differences in CH_4_ yields were driven by a reduction in the control group’s DMI in the respiration chambers compared to DMI in group pens (Table 2).

### 3.2. Performance Recording

There were no differences in mean ADG (control: 0.9 ± 0.22 kg/day, treatment: 0.9 ± 0.33 kg/day) or mean DMI (control: 9.8 ± 0.79 kg/day, treatment: 9.8 ± 1.36 kg/day) during the feed efficiency recording period. None of the variables included in the ANOVA analysis had a significant effect on FCR. The final BWs for the control group were 511 ± 7.1 kg, and for the treatment group, 512 ± 9.3 kg.

## 4. Discussion

A wide range of essential oils and blends of essential oils have been tested for their efficacy in reducing enteric CH_4_. Results have been highly variable, ranging from considerable increases in CH_4_ (e.g., 21% increase [12]) to substantial decreases (e.g., 38% reduction [13]). This variability has been attributed to the type and concentration of active compounds in essential oil, the dose administered, the basal diet, and the length of time supplemented [8,14]. 

Previously published studies assessing the efficacy of AR have focused on lactating dairy cows [15,16,17,18,19], with one study on beef heifers [15]. A meta-analysis found a mean reduction in methane production (gCH_4_ per day) from lactating dairy cows supplemented with AR of 8.8%, but only when supplemented for more than four weeks [7]. Studies where CH_4_ production was measured with less than four weeks of supplementation resulted in inconsistent results. Belanche et al. [7] suggest that a four-week adaptation period is required for AR to be effective at reducing CH_4_ emissions. Castro-Montoya et al. [15] observed numerically lower CH_4_ yield from four Belgian Blue beef heifers (11% decrease) 46 days after the addition of AR to the diet. The lack of significance in the Castro-Montoya et al. [15] study may be due to the low number of animals measured. 

The difference in CH_4_ yield observed between the treatment and control groups in this study was driven by a lower DMI in the control group within the respiration chambers rather than lower CH_4_ production in the treatment group. However, no significant difference in DMI was detected during the performance recording period, and there was no significant effect on FCR. A meta-analysis of studies on lactating dairy cows supplemented with AR found no impact of AR on DMI but a significantly higher feed conversion efficiency [7].

A possible explanation for the reduced DMI in the control animals relative to treatment animals in this experiment could be due to the essential oils having a ‘calming’ effect on the treatment animals. Isolation from conspecifics, such as confinement in a respiration chamber, can cause stress in cattle [20], which leads to reduced feed intake [21]. Llonch et al. [22] found a significant reduction in DMI during the confinement of beef steers in respiration chambers, despite a six-day habituation period in training pens and visual contact with conspecifics whilst in respiration chambers. Although there is no direct evidence that AR reduces stress in cattle, supplementation with oregano essential oil (one of the constituents of AR) has been shown to reduce serum cortisol and norepinephrine concentrations (hormones released during stress) in pigs after transportation [23], and active ingredients found in coriander seed oil (linalool and caryophyllene) reduce anxiety in mice [24].

Dairy cow production has been shown to be favorably affected by AR in some studies (increased milk yield [15,18] and increased fat-corrected milk yield [25]) but not universally (no effect on milk yield [26] and no effect on energy-corrected milk yield [18]). 

Adaptation of the rumen microbiome to essential oils has been identified as a key problem in their use as a CH_4_ mitigation strategy [27]. Although the lower CH_4_ yield in animals receiving AR was driven by reduced DMI by the control group whilst, in respiration chambers, the effect was observed at both 46 and 116 days after AR introduction. Hart et al. [18] found that lactating dairy cows produced significantly less enteric CH_4_ on an absolute (g/day) and yield (g/kg milk) basis. Methane measurements were taken throughout the period using a GreenFeed system, with differences in methane yield observed between four and 21 weeks of a 22-week period.

This study has highlighted the potential implications of isolating cattle in respiration chambers, and future research could explore the possible anxiolytic effects of AR on cattle.

## 5. Conclusions

Although no reduction in enteric CH_4_ production was detected in this study after supplementation with AR, the treatment group did have a significantly lower CH_4_ yield. This was driven by a drop in DMI by control animals while isolated in respiration chambers. However, no difference in DMI between the control and treatment groups was observed in group pens.

## Figures and Tables

**Table 1:** Daily fresh weight intakes and DMI were recorded for each animal.

Item.	Control	Treatment
Components (g/kg DM)		
Grass Silage	489	486
Barley	284	281
Dark Grains	194	189
Molasses	24	28
Minerals	9	16
Composition (g/kg DM)		
Ash (g/kg DM)	76.5	78.8
Dry matter (g/kg)	383	378
Crude protein (g/kg DM)	173	178
AHEE (g/kg DM)	40.5	37.5
NDF (g/kg DM)	361	359
NCGD (% by Wt DM)	81.9	80.0
Starch (g/kg DM)	154	149
Metabolizable energy (MJ/kg DM)	12.5	12.1

DM: dry matter, AHEE: acid hydrolyzed ether extract, NDF: neutral detergent fiber, NCGD: neutral cellulase gammanase digestibility.

**Table 2:** Results from ANOVA of dry matter intake (DMI), daily methane production, and methane yield.

Response Variable	Time Point (Days)	Control Mean	Treatment Mean	Variable Significance (*p*)			
				Block	Chamber	Baseline Covariate	Treatment
DMI (kg)	−3	9.0 (0.05)	9.4 (0.05)	-	-	-	-
	46	9.1 (0.05)	9.8 (0.05) *	<0.01	ns	ns	<0.05
	116	8.7 (0.05)	9.9 (0.08) *	ns	ns	ns	<0.05
CH_4_ (g/day)	−3	206 (2.1)	209 (2.1)	-	-	-	-
	46	223 (2.1)	224 (2.1)	ns	ns	<0.01	ns
	116	217 (2.1)	218 (2.4)	ns	ns	<0.01	ns
CH_4_ yield (g/kg DMI)	−3	23.1 (0.24)	22.2 (0.17)	-	-	-	-
	46	24.7 (0.22)	22.8 (0.19) *	<0.01	<0.05	<0.05	<0.05
	116	25.5 (0.21)	22.7 (0.19) *	<0.05	<0.05	ns	<0.01

Group means and significance of variables included in linear models at −3, 46, and 116 days relative to the introduction of Agolin Ruminant. * = significant difference between group means (*p* < 0.05), ns = not significant.

## Data Availability

Data is available upon request.
